# ASSOCIATIONS OF PERCEIVED PHYSICAL AND MENTAL FATIGABILITY WITH COGNITIVE PERFORMANCE

**DOI:** 10.1093/geroni/igad104.1254

**Published:** 2023-12-21

**Authors:** Benjamin Schumacher, Caterina Rosano, Yujia (Susanna) Qiao, Andrea Rosso, Peggy Cawthon, Stephen Kritchevsky, Nancy Glynn

**Affiliations:** University of Pittsburgh School of Public Health, Pittsburgh, Pennsylvania, United States; University of Pittsburgh, Pittsburgh, Pennsylvania, United States; University of Pittsburgh, Pittsburgh, Pennsylvania, United States; University of Pittsburgh, Pittsburgh, Pennsylvania, United States; California Pacific Medical Center, Research Institute, San Francisco, California, United States; Wake Forest School of Medicine, Winston Salem, North Carolina, United States; University of Pittsburgh School of Public Health, Pittsburgh, Pennsylvania, United States

## Abstract

Greater perceived fatigability has been associated with neurological diseases, but we do not know whether there are associations with cognition among at-risk older adults. At baseline, SOMMA participants completed the Pittsburgh Fatigability Scale (PFS) Physical and Mental subscales (each range 0–50; higher scores = greater fatigability; clinically meaningful increment=4-point physical, 3-point mental) and four cognitive function assessments [Digit Symbol Substitution Test (DSST), Montreal Cognitive Assessment (MoCA), Trail Making Test Part B (TMT-B), and California Verbal Learning Test-Second Edition, Short Form (CVLT-II SF)]. Linear regression quantified associations between PFS subscales and cognitive assessment scores adjusting for site, age, sex, race, education, marital status, and history of stroke, cancer, heart failure, and lung disease. In the 873 participants (59.2% women; age 76.3+/-5.0 years; 85% White), 54% had greater physical fatigability (PFS Physical≥15) and 23% had greater mental fatigability (PFS Mental≥13). Prevalence of cognitive impairment was 2% moderate (MoCA 10-17) and 0% severe ( <10). After adjustments, for each 4-point higher PFS Physical score participants had 0.8 fewer correct DSST items [Beta coefficient and 95% confidence interval: -0.8 (-1.2, -0.4); n=866] and 2.0 seconds slower TMT-B time [2.0 (0.1, 3.8); n=835]. Associations were similar for each 3-point higher PFS Mental score [DSST: -0.7 (-1.1, -0.4) and TMT-B time: 2.5 (1.0, 4.1)]. Neither PFS subscale was associated with MoCA or CVLT. Our results suggest that higher perceived physical and mental fatigability scores may both be indicative of cognitive impairment, particularly in processing speed.

